# *Banhasasim-tang* Attenuates Corticosterone-Induced Behavioral Despair-like Responses and Is Associated with Gut Microbiota and Serum Metabolomic Changes in Mice

**DOI:** 10.4014/jmb.2606.06018

**Published:** 2026-08-04

**Authors:** Seung-Ho Seo, Sang-Mi Kang, Siwon Kang, Yanghee You, Yujin Lee, Mee-Hyun Lee, Chang-Su Na

**Affiliations:** College of Korean Medicine, Dongshin University, 120-9 Dongsindae-gil, Naju 58245, Republic of Korea

**Keywords:** Korean medicine, Banhasasim-tang, Depression, Multiomics

## Abstract

*Banhasasim-tang* (BHSST) is a classical East Asian herbal formula traditionally used for gastrointestinal disorders, but its effects on stress-related depressive-like behavior and the associated microbiome–metabolome signatures remain unclear. This study evaluated the antidepressant-like effects of BHSST in a corticosterone (CORT)-induced mouse model and investigated fecal microbiota and serum metabolomic alterations. Male C57BL/6 mice were assigned to Normal, CORT, fluoxetine, low-dose BHSST, or high-dose BHSST groups. CORT was administered for 21 days, and immobility-based behavioral responses were assessed using the forced swimming test and tail suspension test. Fecal microbiota were analyzed by 16S rRNA gene sequencing, and serum metabolites were profiled using untargeted UPLC-QTOF-MS. BHSST reduced CORT-induced immobility time in both behavioral tests. Although alpha diversity was not significantly altered, BHSST treatment was associated with changes in fecal microbial community structure and discriminative bacterial taxa. Serum metabolomics revealed BHSST-H-associated changes in metabolites related to amino acid metabolism, energy metabolism, redox-related metabolism, lipid metabolism, and microbial co-metabolism. Integrated microbiome-metabolome analysis identified association-based multi-omics signatures involving selected bacterial taxa, serum metabolites, and immobility-based behavioral outcomes. These findings suggest that BHSST-induced behavioral improvement is accompanied by changes in the microbiome–metabolome profile in CORT-induced mice.

## Introduction

Major depressive disorder is a complex neuropsychiatric disorder characterized by persistent mood disturbance, reduced motivation, cognitive and somatic symptoms, and impaired quality of life [1]. Although monoaminergic dysfunction has long been considered a major biological mechanism of depression, accumulating evidence indicates that depression involves broader systemic alterations, including hypothalamic–pituitary–adrenal axis dysregulation, neuroinflammation, oxidative stress, metabolic disturbance, and microbiota–gut–brain axis dysfunction [1, 2]. Prolonged activation of the hypothalamic–pituitary–adrenal axis and excessive glucocorticoid exposure are closely linked to stress-related depressive symptoms [2].

The gut microbiota is increasingly recognized as a key biological interface between environmental stress, host metabolism, and brain function [3, 4]. Gut microorganisms can influence host neurobiology through immune regulation, neuroendocrine signaling, microbially derived metabolites, tryptophan metabolism, short-chain fatty acid production, bile acid metabolism, and neurotransmitter-related pathways [3, 4]. Altered gut microbial composition has been reported in patients with depression, and experimental microbiome remodeling has been shown to induce depressive-like behaviors through host metabolic pathways [5, 6]. In parallel, peripheral blood metabolomic studies in patients with major depressive disorder have identified alterations in tryptophan–kynurenine metabolism, fatty acid metabolism, amino acid metabolism, and other metabolic pathways [7]. Therefore, integrated analysis of fecal microbiota and serum metabolomics may provide a useful framework for identifying microbial–metabolic signatures associated with depressive-like behavior and treatment response [8].

*Banhasasim-tang* (BHSST), corresponding to *Banxia Xiexin* decoction in Chinese medicine, is a classical East Asian herbal formula traditionally used for gastrointestinal disorders [9]. The BHSST preparation used in this study was based on the Korean formulation and consisted of *Pinelliae Tuber*, *Scutellariae Radix*, *Ginseng Radix*, *Glycyrrhizae Radix* et *Rhizoma*, *Zingiberis Rhizoma*, *Coptidis Rhizoma*, *Zingiberis Rhizoma Recens*, and *Zizyphi Fructus*. Although BHSST has mainly been studied in gastrointestinal and inflammatory disorders, previous studies suggest that it may also have biological relevance to neural and stress-related conditions. BHSST has been reported to protect hippocampal neurons against oxidative stress and improve cognitive dysfunction in experimental models [10]. Network pharmacology analysis has also suggested that *Banxia Xiexin* decoction may target biological processes shared by depression and ulcerative colitis, including inflammatory response, oxidative stress response, steroid metabolism, lipid metabolism, serotonergic synapse signaling, arachidonic acid metabolism, HIF-1 signaling, and NF-κB signaling [11]. In addition, microbiome- and metabolomics-based studies have reported that *Banxia Xiexin* decoction modulates the gut microbiome–lipid metabolic axis in an atherosclerosis co-depression model and regulates gut microbiota-associated metabolism in intestinal inflammation models [12, 13]. However, whether BHSST reduces corticosterone (CORT)-induced immobility-based behavioral responses and how these changes are associated with fecal microbiota and serum metabolomic alterations remain insufficiently understood.

Therefore, the present study aimed to evaluate the antidepressant-like effects of BHSST in a CORT-induced mouse model and to characterize the associated microbial and metabolic changes. Male C57BL/6 mice were exposed to CORT and treated with fluoxetine or BHSST at two doses. Antidepressant-like activity was evaluated using the forced swimming test and tail suspension test, which assess immobility-based behavioral responses in rodents [14, 15]. Fecal microbiota were analyzed using 16S rRNA gene sequencing, and serum metabolic profiles were analyzed using untargeted ultra-performance liquid chromatography–quadrupole time-of-flight mass spectrometry (UPLC-QTOF-MS)-based metabolomics. By integrating behavioral, microbiome, and metabolomic datasets, this study sought to identify gut microbial–metabolic signatures associated with BHSST treatment and to provide microbiological and metabolic context for BHSST-associated changes in a stress-related depression-like model.

## Materials and Methods

### Animals and Experimental Design

Seven-week-old male C57BL/6 mice weighing 19–21 g were purchased from Orient Bio Inc. (Republic of Korea). The animals were maintained under specific pathogen-free conditions in a controlled room at 20 ± 2°C and 60 ± 5% relative humidity with a 12 h light/dark cycle. Food and water were provided ad libitum. After a 2-week acclimatization period, the mice were randomly divided into five groups with eight mice per group: Normal, CORT, FLX, BHSST-L, and BHSST-H.

The CORT group received CORT at 20 mg/kg/day. The FLX group received CORT and fluoxetine at 15 mg/kg/day. The BHSST-L and BHSST-H groups received CORT and BHSST at 100 and 300 mg/kg/day, respectively. CORT exposure and drug treatment were performed concurrently for 21 consecutive days. The forced swimming test was conducted on day 20, and the tail suspension test was conducted on day 21. Blood and fecal samples were collected on the day after the tail suspension test. All animal procedures were approved by the Institutional Animal Care and Use Committee of Dongshin University under approval number DSU2025-04-02.

### Preparation and Administration of BHSST

BHSST was purchased from KBPharm Inc. (Republic of Korea). The formula was based on the Korean BHSST preparation and consisted of *Pinelliae Tuber* 2.5 g, *Scutellariae Radix* 1.88 g, *Ginseng Radix* 1.88 g, *Glycyrrhizae Radix* et *Rhizoma* 1.88 g, *Zingiberis Rhizoma* 1.25 g, *Coptidis Rhizoma* 0.63 g, *Zingiberis Rhizoma Recens* 0.5 g, and *Zizyphi Fructus* 0.67 g. Because a commercially available preparation was used, no additional extraction procedure was performed in this study. BHSST was prepared in distilled water and orally administered once daily at doses of 100 or 300 mg/kg/day according to body weight.

### Corticosterone and Fluoxetine Treatment

CORT and fluoxetine were purchased from Sigma-Aldrich (USA). CORT was dissolved in corn oil and administered by subcutaneous injection at 20 mg/kg/day for 21 consecutive days to induce depressive-like behavior. Fluoxetine was dissolved in phosphate-buffered saline and orally administered at 15 mg/kg/day as a positive control drug. BHSST was orally administered during the same experimental period. The Normal group received vehicle treatment instead of corticosterone and active drugs.

### Behavioral Test

The forced swimming test was performed on day 20 to evaluate depression-like behavioral responses. Each mouse was individually placed in a transparent glass cylinder measuring 30 cm in height and 18 cm in diameter. The cylinder was filled with water to a depth of 20 cm, and the water temperature was maintained at 24 ± 2°C. Each trial lasted for 6 min. The first 2 min were considered an adaptation period, and immobility time was analyzed during the final 4 min. Immobility was defined as the absence of active escape-directed movement, except for minimal motions required to keep the head above the water. After each trial, the mouse was removed from the cylinder, dried, and returned to its home cage. The water was replaced between trials. Immobility time was quantified using SMART video tracking software version 3.0 (Panlab, Harvard Apparatus, USA).

The tail suspension test was conducted on day 21. Each mouse was suspended by the tail using adhesive tape attached approximately 1 cm from the tail tip. The mouse was placed in an individual testing chamber measuring 25 × 25 × 30 cm, with the body suspended above the floor. Each session lasted for 6 min. The first 1 min was considered an adaptation period, and immobility time was analyzed during the remaining 5 min. Immobility was defined as the absence of active body movement while the mouse remained passively suspended. The test was conducted under quiet and consistent environmental conditions. Immobility time was quantified using SMART video tracking software version 3.0 (Panlab). Behavioral video analysis for both the FST and TST was performed by an investigator blinded to the treatment group allocation.

### Serum and Fecal Sample Collection

Sample collection was performed on the day after the tail suspension test. Mice were anesthetized with isoflurane, and blood samples were collected by cardiac puncture. Serum was separated by centrifugation at 3,500 rpm for 10 min at 4°C and stored at -80°C until metabolomic analysis. Fecal samples were collected from residual fecal material in the colon after sacrifice. Each fecal sample was collected individually and stored at -80°C until microbial DNA extraction and 16S rRNA gene sequencing.

### 16S rRNA Gene Sequencing and Gut Microbiota Analysis

Microbial DNA was extracted from fecal samples using the AllEx™ Mini Automated Nucleic Acid Extraction System (GeneAll Biotechnology Co., Ltd., Republic of Korea) according to the manufacturer’s instructions. The V4 region of the bacterial 16S rRNA gene was amplified using V4-specific primers containing Illumina overhang adapter sequences. Indexed libraries were prepared using a limited-cycle amplification step, normalized, pooled, and sequenced on the Illumina MiSeq platform using v3 reagents.

Raw sequence reads were assessed using FastQC version 0.12.1. Denoising, quality filtering, paired-end read merging, and amplicon sequence variant generation were performed using DADA2 within QIIME2 version 2023.2. Taxonomic assignment was performed using a SILVA-based classifier. For the samples included in the present study, raw sequencing reads ranged from 61,828 to 126,984 reads per sample, and merged non-chimeric reads after quality filtering ranged from 20,690 to 45,885 reads per sample. Alpha and beta diversity analyses were performed after rarefaction to 20,559 reads per sample. The number of observed ASVs ranged from 222 to 397 per sample. Rarefaction curves for observed features, Chao1, Shannon, and Simpson indices approached a plateau at the rarefied sequencing depth, indicating adequate sequencing depth for diversity analysis. The feature table was converted to relative abundance for downstream community analysis. Alpha diversity was evaluated using Chao1, Shannon, and Simpson indices. Beta diversity was assessed using Bray–Curtis dissimilarity based on genus-level relative abundance and visualized by principal coordinate analysis. Differences in microbial community structure among groups were tested using PERMANOVA with 999 permutations. Differentially enriched bacterial taxa were identified using linear discriminant analysis effect size (LEfSe), and taxa satisfying *p* < 0.05 and a linear discriminant analysis (LDA) score ≥ 2.0 were considered discriminative taxa.

### Serum Sample Preparation for Untargeted Metabolomics

Serum samples were prepared using organic solvent-based protein precipitation. Briefly, 100 μL of serum was mixed with 400 μL of extraction solvent consisting of acetonitrile and methanol at a 3:1 ratio. The mixture was vortexed for 1 min and extracted at 4°C for 1 h. After extraction, the samples were centrifuged at 14,500 rpm for 15 min at 4°C. The supernatant was collected and used for UPLC-QTOF-MS analysis. Quality control samples were prepared by pooling 20 μL aliquots from each extracted sample and were used to monitor analytical stability throughout the LC-MS sequence.

### Serum Metabolomic Profiling by UPLC-QTOF-MS

Serum metabolomic profiling was performed using a Waters ACQUITY UPLC I-Class system coupled to a Xevo G3 QTOF mass spectrometer (Waters Corp., USA). Chromatographic separation was achieved using an ACQUITY UPLC HSS T3 column (2.1 × 100 mm, 1.8 μm). The mobile phase consisted of water containing 0.1% formic acid as solvent A and acetonitrile containing 0.1% formic acid as solvent B. The flow rate was set at 0.4 mL/min, and the injection volume was 2 μL.

The gradient program was as follows: 95% A from 0 to 3 min, 95–50% A from 3 to 8 min, 50–5% A from 8 to 12 min, 5% A from 12 to 14 min, and re-equilibration at 95% A from 14 to 20 min. Mass spectrometric analysis was performed in both positive and negative electrospray ionization modes. The capillary voltage was set to 2.8 kV in positive ion mode and 2.5 kV in negative ion mode. The cone voltage was 40 V. The source temperature was maintained at 120°C, and the desolvation temperature was set at 500°C. The desolvation gas flow rate was 1,000 L/h, and the cone gas flow rate was 150 L/h.

Data were acquired in data-independent MS^E^ mode over a mass range of *m/z* 50–1,200 with a scan time of 0.1 sec. Sodium formate was used for mass calibration. Leucine enkephalin at 200 pg/mL was used as the lock mass for real-time mass correction, with reference ions at *m/z* 556.2771 in positive mode and *m/z* 554.2620 in negative mode. Argon was used as the collision gas, and collision energy was ramped from 20 to 40 eV in the transfer collision cell. To ensure system stability and column equilibration, QC samples were injected before the analytical sequence. During the sequence, a blank sample and a QC sample were injected after every ten study samples.

### Metabolomics Data Processing and Metabolite Annotation

Raw LC-MS data acquired using MassLynx version 4.1 (Waters Corp.) were imported into Progenesis QI software (Nonlinear Dynamics, UK) for untargeted metabolomics processing. QC samples were used as the reference for retention time alignment. Peak detection, deconvolution, alignment, and normalization were performed across all samples. Ion intensities were normalized to the total ion current to reduce analytical variation. Features detected in blank samples were removed before downstream analysis. Analytical reproducibility was assessed using QC samples, and features with a QC relative standard deviation greater than 30% were excluded.

After filtering, zero values were treated as missing or below-detection values and replaced using half of the minimum non-zero value for each feature. The processed intensity matrix was then log2-transformed and Pareto-scaled before multivariate analysis. Positive and negative ion mode datasets were processed and analyzed separately.

Metabolite annotation was performed using accurate mass and MS/MS fragmentation information. Candidate metabolites were assigned using a mass tolerance of 10 ppm and searched against the Human Metabolome Database and the MassBank of North America. Metabolite identification confidence was assigned according to the Metabolomics Standards Initiative (MSI) guidelines. Because authentic reference standards were not analyzed under identical chromatographic and mass spectrometric conditions, the annotated metabolites were classified as MSI Level 2 putatively annotated compounds rather than MSI Level 1 identified metabolites. Unknown features were retained for global multivariate analysis but excluded from metabolite-level biological interpretation.

Principal component analysis (PCA) was performed to visualize overall serum metabolic variation in positive and negative ion modes. Group differences in the processed metabolomics matrix were additionally assessed using permutational multivariate analysis of variance based on Euclidean distance. To identify metabolites contributing to the metabolic difference between the CORT and BHSST-H groups, orthogonal partial least squares discriminant analysis (OPLS-DA) was performed separately for positive and negative ion modes. Model robustness was evaluated using 200 permutation tests. Variable importance in projection (VIP) scores were extracted from the OPLS-DA models. Differential metabolites were selected using VIP ≥ 1.0, *p* < 0.05, and Benjamini–Hochberg FDR *q* < 0.1. For biological interpretation, only annotated metabolites were retained, and unknown features were excluded. Pathway enrichment and topology analysis were performed using MetaboAnalyst 6.0 with the KEGG mouse pathway library. Detailed annotation information for the differential metabolites, including metabolite name, ion mode, *m/z*, retention time, adduct type, mass error, MS/MS evidence, database source, and MSI identification confidence level, is provided in Supplementary Table S1.

### Multi-Omics Integration

Integrated microbiome–metabolome analysis was performed to determine whether BHSST-associated fecal bacterial taxa and serum metabolites formed a coordinated multi-omics signature related to depressive-like behavior. The microbiome block consisted of LEfSe-selected fecal bacterial taxa, and the metabolome block consisted of annotated differential serum metabolites identified from the CORT versus BHSST-H comparison. Microbial abundance values were transformed using log10(relative abundance + pseudo-count), and metabolite intensities were analyzed using the processed log2-transformed matrix.

Block sparse partial least squares discriminant analysis (block.sPLS-DA) was performed using the Data Integration Analysis for Biomarker discovery using Latent cOmponents (DIABLO) framework in the mixOmics R package [16]. Experimental group was used as the response variable. A two-component model was constructed to visualize integrated group separation and to identify microbial and metabolic features contributing to the integrated components. Loading values were extracted from integrated component 1 to identify features contributing to group discrimination. To visualize cross-omics associations among selected features, a microbiota–metabolite network was constructed using DIABLO-selected microbiome and metabolome features. Spearman correlations were calculated between selected bacterial taxa and serum metabolites across matched samples. All selected features were retained as network nodes, and statistically meaningful microbiota–metabolite associations were displayed as edges. Node size represented the absolute loading value, and edge color and thickness represented the direction and magnitude of Spearman correlation, respectively. To examine behavioral relevance, integrated and block-specific component scores were correlated with FST and TST immobility time using Spearman correlation analysis. Group-wise differences in integrated component 1 scores were assessed using Wilcoxon rank-sum tests. This integrative analysis was used to identify statistical associations among microbiome, metabolome, and behavioral features, and was not intended to infer direct biological interactions or causal relationships.

### Statistical Analysis

Statistical analyses were performed using GraphPad Prism and R. Continuous data were assessed for normality using the Shapiro–Wilk test. Homogeneity of variance was evaluated using Brown–Forsythe or Levene’s test when applicable. For normally distributed data with equal variance, group differences were analyzed using one-way analysis of variance followed by Tukey’s multiple comparison test. For data that did not satisfy parametric assumptions, the Kruskal–Wallis test followed by Dunn’s multiple comparison test was used. Behavioral data were analyzed using the Kruskal–Wallis test followed by Dunn’s multiple comparison test.

For microbiome analysis, alpha diversity indices were compared among groups using the Kruskal–Wallis test. Beta diversity was assessed using Bray–Curtis dissimilarity and visualized by principal coordinate analysis. Overall differences in microbial community structure among groups were tested using PERMANOVA with 999 permutations. LEfSe was used to identify discriminative taxa, with *p* < 0.05 and LDA score ≥ 2.0 as selection criteria. For selected LEfSe-derived taxa, global differences among groups were first assessed using the Kruskal-Wallis test. Taxa showing *p* < 0.05 were further evaluated using predefined Dunn’s post hoc comparisons with Benjamini-Hochberg correction. Only significant predefined comparisons were displayed in Fig. 3B. As a sensitivity analysis, group-associated genus-level taxa were additionally evaluated using MaAsLin2 [17]. Relative abundance values were arcsine square-root transformed, the CORT group was used as the reference group, and *p*-values were adjusted using the Benjamini-Hochberg method. The MaAsLin2 results are presented in Supplementary Fig. S1 and Supplementary Table S2.

For metabolomics analysis, PCA was used to visualize global metabolic variation. PERMANOVA was used to test overall group effects in the processed metabolomics matrix. OPLS-DA was used to identify metabolites contributing to the CORT versus BHSST-H separation, and model validity was assessed using 200 permutation tests. Differential metabolites were selected using VIP ≥ 1.0, *p* < 0.05, and FDR *q* < 0.1. Pathway results were interpreted as nominal when raw *p* < 0.05 and exploratory when they did not pass FDR correction.

Spearman correlation analysis was used to evaluate associations among behavioral parameters, bacterial taxa, serum metabolites, and multi-omics component scores. Unless otherwise specified, statistical significance was set at *p* < 0.05.

## Results

### BHSST Reduced Corticosterone-Induced Behavioral Despair-Like Responses in Mice

**Fig. 1:**
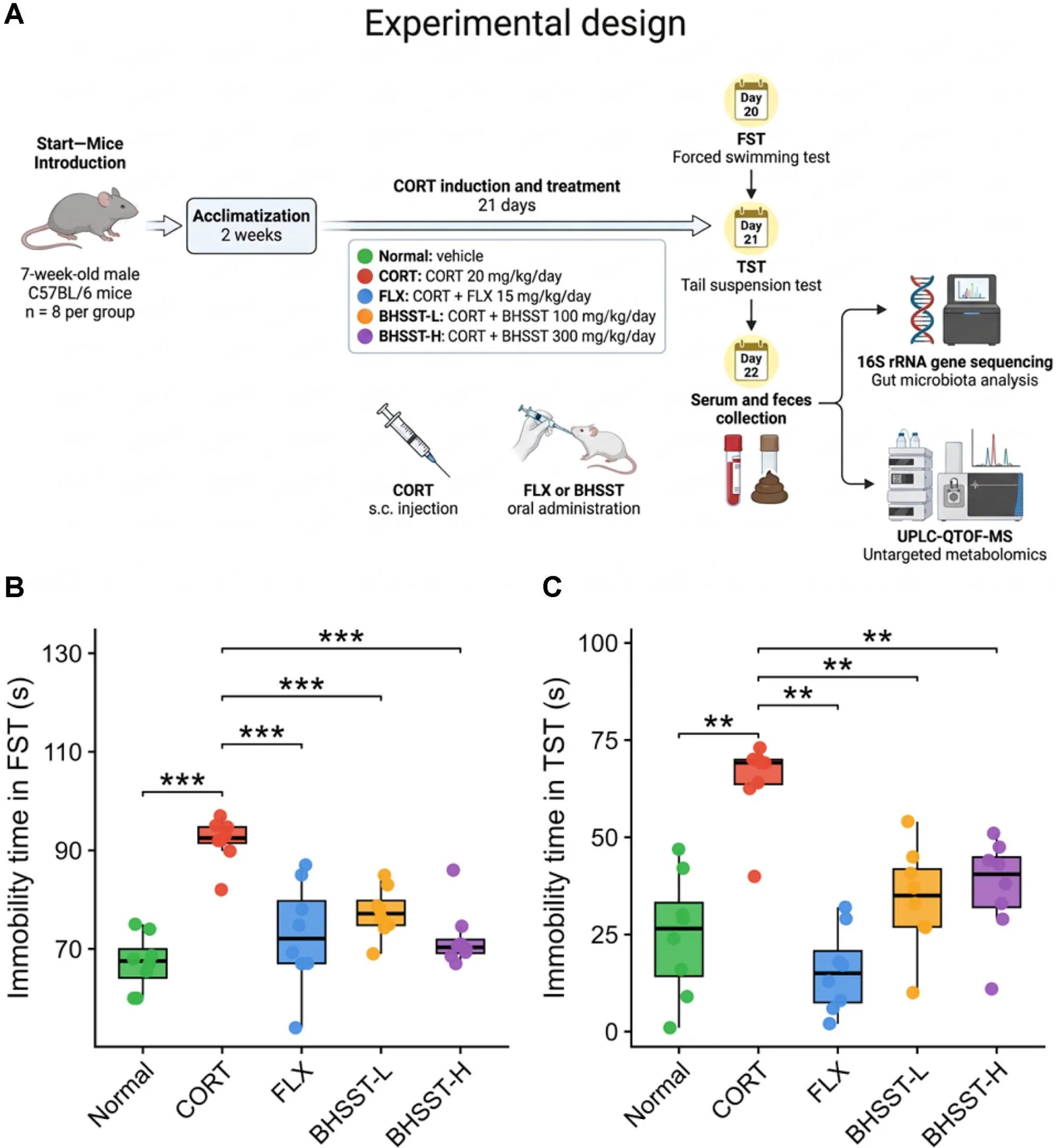
Experimental design and behavioral effects of *Banhasasim-tang* in corticosterone-induced mice. (**A**) Experimental design. Seven-week-old male C57BL/6 mice were acclimatized for 2 weeks and subjected to CORT exposure with or without fluoxetine (FLX) or BHSST treatment for 21 days. The forced swimming test (FST) and tail suspension test (TST) were conducted on days 20 and 21, respectively. Serum and fecal samples were collected on day 22 for 16S ribosomal RNA (16S rRNA) gene sequencing and ultra-performance liquid chromatography-quadrupole time-of-flight mass spectrometry (UPLC-QTOF-MS)-based metabolomics. (**B**) FST immobility time. (**C**) TST immobility time. Behavioral video analysis for both tests was performed by an investigator blinded to the treatment group allocation. Box plots show individual data points. Statistical significance was assessed using the Kruskal–Wallis test followed by Dunn’s multiple comparison test. **p < 0.01; ***p < 0.001. CORT, corticosterone; FLX, fluoxetine; BHSST-L, low-dose *Banhasasim-tang*; BHSST-H, high-dose *Banhasasim-tang*.

The experimental design is summarized in Fig. 1A. Seven-week-old male C57BL/6 mice were exposed to CORT for 21 consecutive days and treated with fluoxetine (FLX) or BHSST during the same period. The forced swimming test (FST) and tail suspension test (TST) were performed on days 20 and 21, respectively, followed by serum and fecal sample collection for subsequent omics analyses.

In the FST, the CORT group showed a significant increase in immobility time compared with the Normal group, confirming the induction of depressive-like behavior (Fig. 1B). FLX treatment significantly reduced CORT-induced immobility. Notably, both low-dose BHSST and high-dose BHSST also significantly decreased immobility time compared with the CORT group, indicating that BHSST attenuated CORT-induced behavioral despair-like responses.

Consistent with the FST results, the TST showed that CORT exposure significantly increased immobility time relative to the Normal group (Fig. 1C). FLX markedly reduced immobility time compared with the CORT group. Both BHSST-L and BHSST-H groups also exhibited significantly lower immobility time than the CORT group. These results indicate that BHSST treatment reduced CORT-induced immobility-based behavioral despair-like responses in the FST and TST.

### BHSST Modulated Fecal Microbial Community Structure in Corticosterone-Induced Mice

**Fig. 2:**
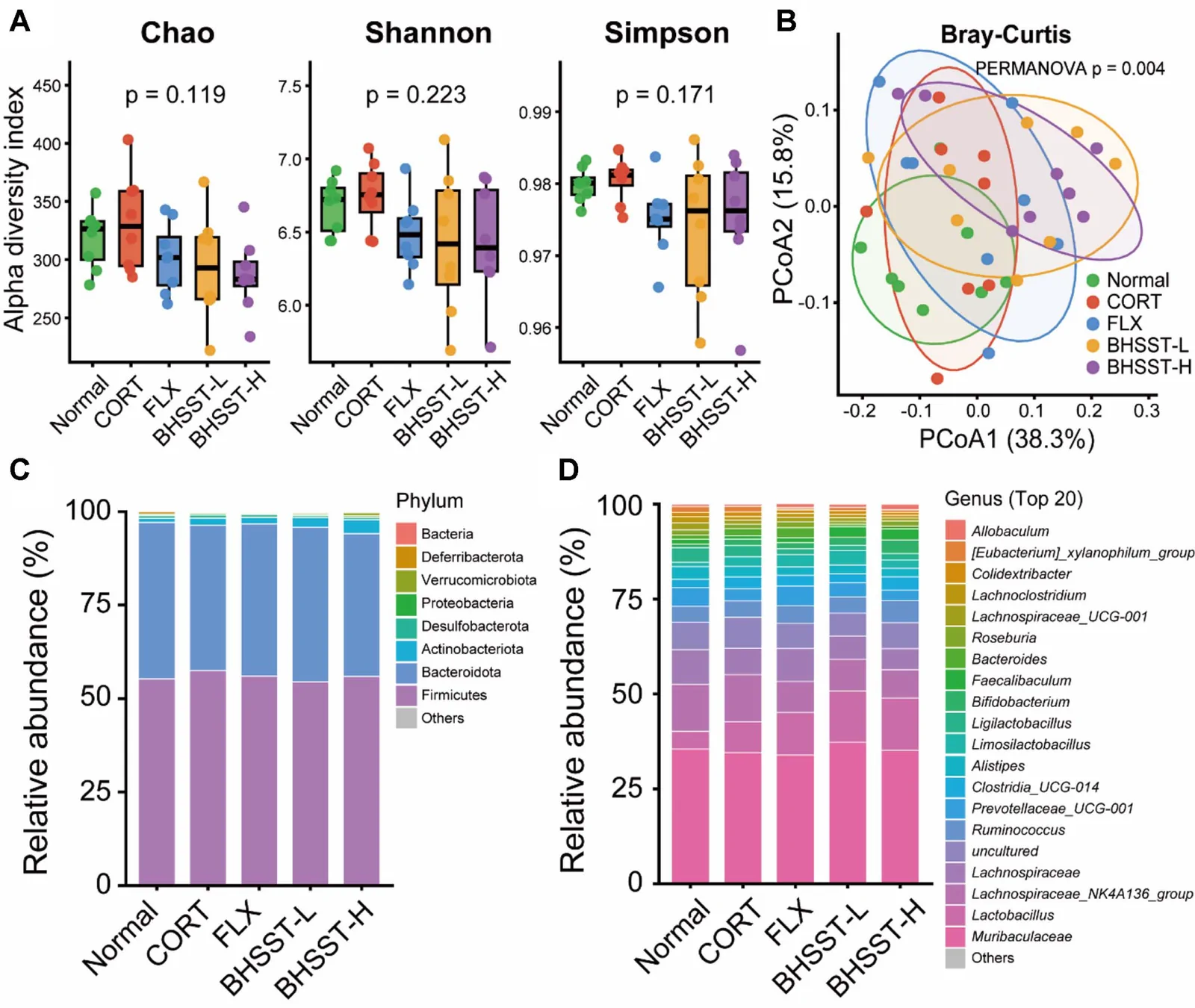
Effects of *Banhasasim-tang* on fecal microbiota diversity and taxonomic composition in corticosterone-induced mice. (**A**) Alpha diversity of fecal microbiota was evaluated using Chao1, Shannon, and Simpson indices. Global *p*-values were calculated using the Kruskal-Wallis test. (**B**) Beta diversity was assessed using Bray-Curtis dissimilarity based on genus-level relative abundance and visualized by principal coordinate analysis (PCoA). Overall differences in microbial community structure among groups were evaluated using permutational multivariate analysis of variance (PERMANOVA). (**C**) Phylum-level taxonomic composition of fecal microbiota. (**D**) Genus-level or lowest assigned taxonomic composition showing the top 20 taxa and Others. Data in (**A**) are shown as box plots with individual data points. CORT, corticosterone; FLX, fluoxetine; BHSST-L, low-dose *Banhasasim-tang*; BHSST-H, high-dose *Banhasasim-tang*.

Fecal microbiota profiles were analyzed using 16S rRNA gene sequencing. Chao1, Shannon, and Simpson indices did not differ significantly among the groups, although all three alpha diversity indices showed a similar pattern, with a tendency to increase in the CORT group and to shift toward the Normal group after FLX or BHSST treatment (Fig. 2A). Bray-Curtis dissimilarity based on genus-level relative abundance revealed group-dependent differences in fecal microbial community structure, and PERMANOVA confirmed a significant overall difference among the experimental groups (*p* = 0.004; Fig. 2B). Phylum-level composition showed that Bacteroidota and Firmicutes were the dominant phyla across all groups (Fig. 2C). Genus-level or lowest assigned taxonomic composition further showed group-dependent differences in dominant taxa, including *Muribaculaceae*, *Lactobacillus*, *Lachnospiraceae_NK4A136*_group, and *Lachnospiraceae* (Fig. 2D). These findings suggest that BHSST treatment was associated with changes in fecal microbial community structure and taxonomic composition in CORT-induced mice.

### BHSST Altered LEfSe-Defined Fecal Bacterial Markers in CORT-Induced Mice

**Fig. 3:**
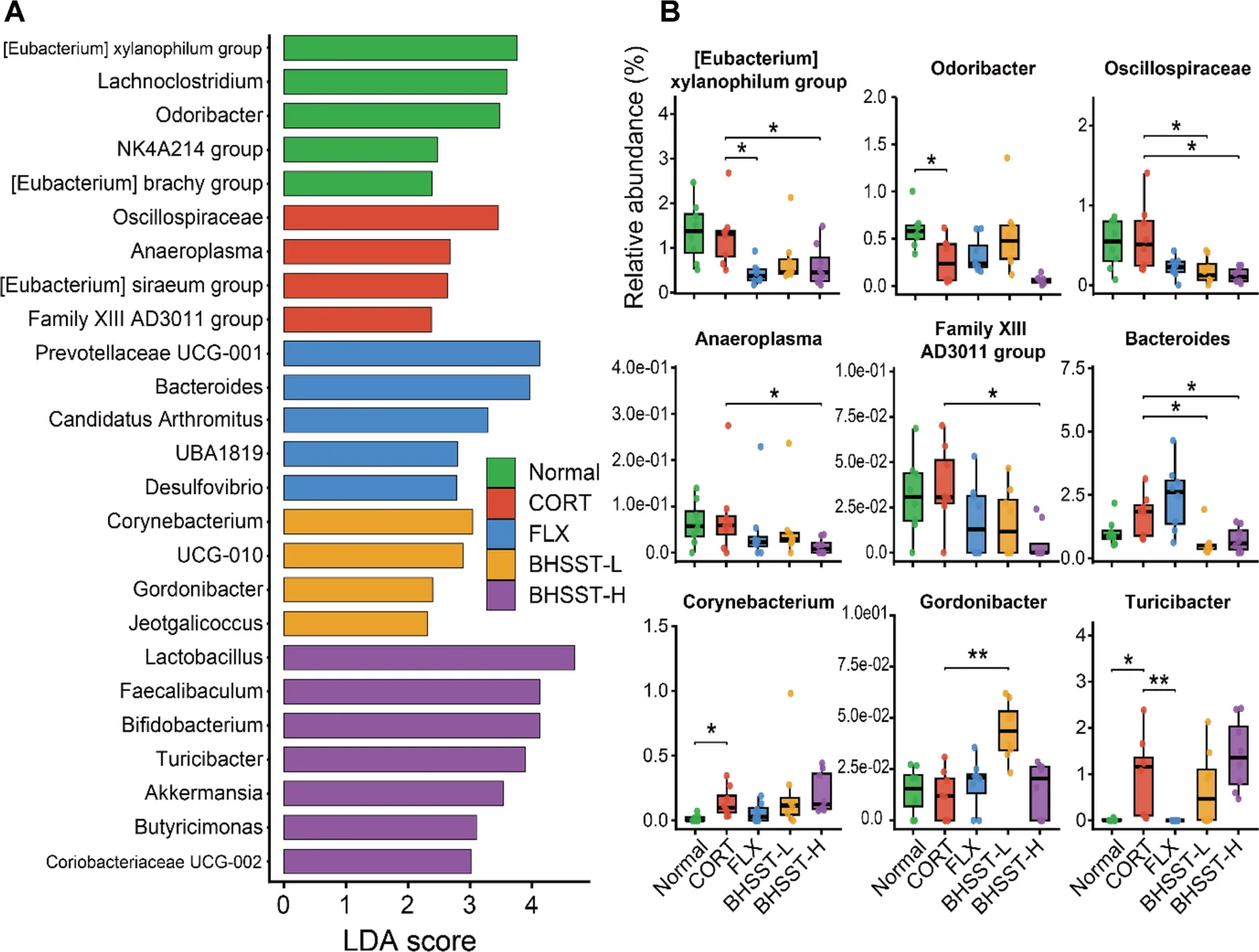
LEfSe-defined fecal bacterial markers in CORT-induced mice treated with *Banhasasim-tang*. (**A**) Linear discriminant analysis effect size (LEfSe) analysis showing differentially enriched fecal bacterial taxa among the experimental groups. Taxa with *p* < 0.05 and an LDA score ≥ 2.0 were considered discriminative markers. (**B**) Raw relative abundance plots of statistically significant LEfSe-derived taxa. Only taxa showing a significant Kruskal-Wallis test result and at least one significant predefined Dunn’s post hoc comparison are displayed. Data are shown as box plots with individual data points. Asterisks indicate significant Dunn’s post hoc comparisons after Benjamini-Hochberg correction: *p < 0.05, **p < 0.01. Free y-axis scales were used for individual taxa, and scientific notation was applied to y-axis tick labels where appropriate to improve the readability of low-abundance taxa. The predefined comparisons were Normal versus CORT, CORT versus FLX, CORT versus BHSST-L, and CORT versus BHSST-H. CORT, corticosterone; FLX, fluoxetine; BHSST-L, low-dose *Banhasasim-tang*; BHSST-H, high-dose *Banhasasim-tang*.

LEfSe analysis was performed to identify bacterial taxa that discriminated among the experimental groups based on statistical significance and effect size. Taxa with *p* < 0.05 and an LDA score ≥ 2.0 were defined as discriminative markers, with higher LDA scores indicating a greater contribution to group differentiation. LEfSe detected group-specific bacterial markers in the Normal, CORT, FLX, BHSST-L, and BHSST-H groups (Fig. 3A). The CORT group was enriched with *Oscillospiraceae*, [Eubacterium] siraeum group, *Anaeroplasma*, and Family XIII AD3011 group. In contrast, the BHSST-H group was enriched with *Lactobacillus*, *Bifidobacterium*, *Faecalibaculum*, *Turicibacter*, *Akkermansia*, *Butyricimonas*, and Coriobacteriaceae UCG-002.

To confirm whether these LEfSe-derived markers showed measurable abundance differences, raw relative abundance values were compared among groups. Taxa with a significant Kruskal-Wallis test result and at least one significant predefined Dunn’s post hoc comparison were visualized (Fig. 3B). The plotted taxa showed significant group-dependent differences in relative abundance. These results indicate that BHSST treatment altered specific fecal bacterial markers in CORT-induced mice. MaAsLin2 sensitivity analysis further identified group-associated taxa relative to the CORT group, although these taxa did not completely overlap with the LEfSe-derived markers (Supplementary Fig. S1 and Table S2).

### BHSST Modulated Serum Metabolic Profiles and Pathway-Level Metabolic Signatures in Corticosterone-Induced Mice

**Fig. 4:**
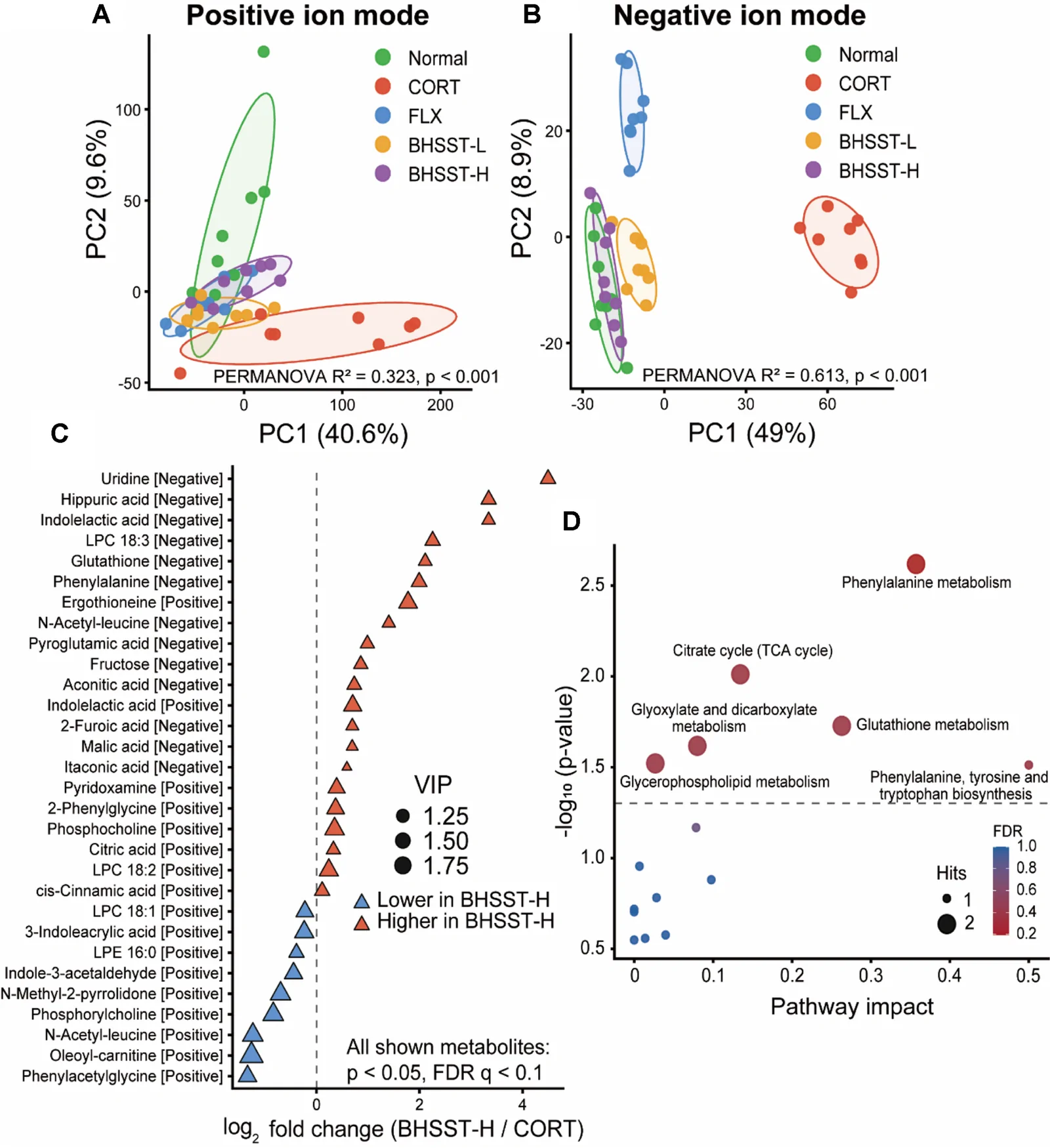
Serum metabolomic profiling and pathway-level interpretation of *Banhasasim-tang*-associated metabolic changes in corticosterone-induced mice. (**A, B**) Principal component analysis (PCA) score plots of serum metabolomic profiles in positive ion mode (**A**) and negative ion mode (**B**). PCA was performed using the processed metabolomics matrix after zero-value handling, half-minimum imputation, log2 transformation, and Pareto scaling. Permutational multivariate analysis of variance (PERMANOVA) R^2^ and p-values were calculated using Euclidean distance based on the processed metabolomics matrix and indicate overall group effects. (**C**) Variable importance in projection (VIP)-based lollipop/dot plot of annotated differential serum metabolites identified from the CORT versus BHSST-H comparison. The x-axis indicates log2 fold change (BHSST-H/CORT). Point size represents VIP score, and color indicates the direction of change. All shown metabolites satisfied *p* < 0.05 and false discovery rate (FDR) *q* < 0.1. Unknown features were excluded from metabolite-level interpretation. (**D**) Pathway enrichment and topology analysis of annotated differential serum metabolites selected from the CORT versus BHSST-H comparison. The x-axis indicates pathway impact, and the y-axis indicates -log10 raw *p*-value. Point size represents the number of matched metabolites, and color indicates the FDR-adjusted value. The dashed line indicates raw *p* = 0.05. Pathways are interpreted as nominally enriched because no pathway passed FDR *q* < 0.1. Detailed metabolite annotation information is provided in Supplementary Table 1. CORT, corticosterone; FLX, fluoxetine; BHSST-L, low-dose *Banhasasim-tang*; BHSST-H, high-dose *Banhasasim-tang*.

Serum metabolomic profiling was performed using untargeted UPLC-QTOF-MS in positive and negative ion modes. Principal component analysis (PCA) revealed group-dependent shifts in serum metabolic profiles in both ion modes (Fig. 4A and 4B). In positive ion mode, the CORT group showed partial separation from the other groups along PC1, and PERMANOVA supported a significant group effect (R^2^ = 0.323, *p* < 0.001; Fig. 4A). In negative ion mode, the CORT group was more clearly separated from the Normal, BHSST-L, and BHSST-H groups, with a stronger group effect supported by PERMANOVA (R^2^ = 0.613, *p* < 0.001; Fig. 4B). These results indicate that CORT exposure markedly altered the serum metabolic profile and that BHSST treatment was associated with a shift in the metabolic state.

To identify metabolites contributing to the metabolic difference between the CORT and BHSST-H groups, OPLS-DA was used to model group-related metabolic separation. VIP scores, which indicate the contribution of individual features to this separation, were extracted from the OPLS-DA model. Differential features were selected using VIP ≥ 1.0, *p* < 0.05, and FDR *q* < 0.1. Among these features, MSI Level 2 putatively annotated metabolites were retained for metabolite-level biological interpretation, whereas unknown features were excluded. The VIP-based lollipop/dot plot showed both increased and decreased putatively annotated features in the BHSST-H group compared with the CORT group (Fig. 4C). These features included metabolites related to amino acid metabolism, gut microbial co-metabolism, lipid metabolism, redox-related metabolism, and energy metabolism. Features annotated as N-acetyl-leucine were detected in both positive and negative ion modes with different directions; therefore, they were treated as mode-specific putatively annotated features and are reported with ion-mode information in Supplementary Table 1.

Pathway enrichment and topology analysis were then performed using the MSI Level 2 putatively annotated differential metabolites from the CORT versus BHSST-H comparison. Several pathways showed nominal enrichment based on raw *p*-values, including phenylalanine metabolism, the citrate cycle, glutathione metabolism, glyoxylate and dicarboxylate metabolism, glycerophospholipid metabolism, and phenylalanine, tyrosine, and tryptophan biosynthesis (Fig. 4D). Among these, phenylalanine metabolism showed the lowest raw *p*-value and a relatively high pathway impact, whereas the citrate cycle and glutathione metabolism were represented by multiple matched metabolites. However, no pathway passed the FDR *q* < 0.1 threshold; therefore, these pathway-level findings were interpreted as exploratory and used to guide biological interpretation rather than as definitive pathway enrichment.

Together, these findings suggest that BHSST-H treatment was associated with broad serum metabolic remodeling in CORT-induced mice, involving changes in amino acid metabolism, energy metabolism, antioxidant-related metabolism, lipid metabolism, and gut microbial co-metabolic signatures.

### Integrated Microbiome–Metabolome Signatures Were Associated with Behavioral Outcomes in CORT-Induced Mice

**Fig. 5:**
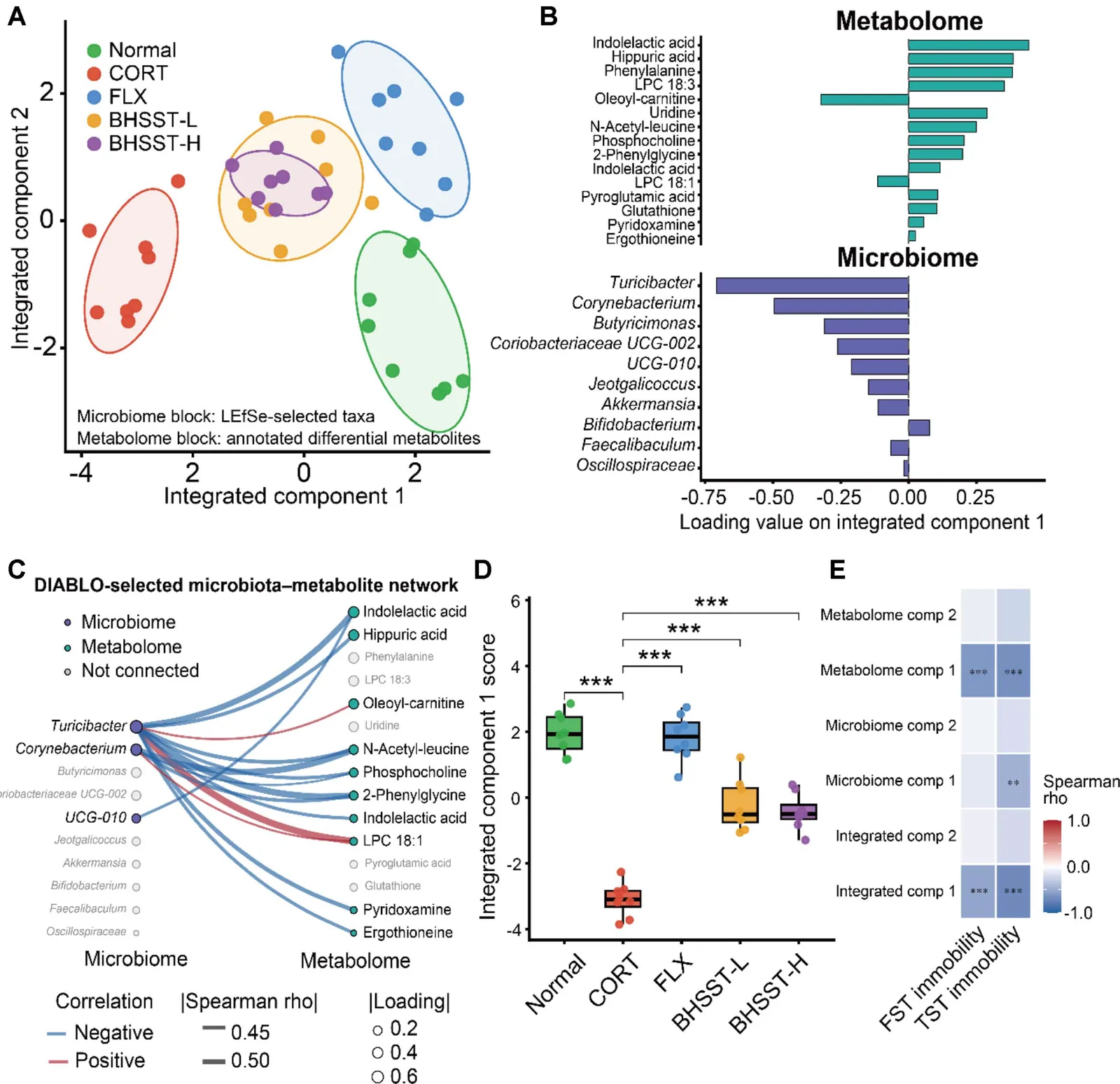
Integrated microbiome–metabolome signatures associated with behavioral outcomes in CORT-induced mice treated with *Banhasasim-tang*. (**A**) Integrated microbiome–metabolome block sparse partial least squares discriminant analysis (block.sPLS-DA) score plot. The microbiome block consisted of LEfSe-selected fecal bacterial taxa, and the metabolome block consisted of MSI Level 2 putatively annotated differential serum metabolites. Each point represents an individual mouse, and ellipses indicate 68% confidence regions for each group. (**B**) Loading plot showing microbiome and metabolome features contributing to integrated component 1. Bar length indicates the loading value, and positive and negative loadings represent opposite directions along the integrated component rather than biological activation or suppression. (**C**) DIABLO-selected microbiota–metabolite association network. All selected features are shown as nodes, and node size represents the absolute loading value. Nodes without significant cross-omics correlations are shown in gray. Edges indicate significant Spearman correlations between selected microbiome and metabolome features, with edge color indicating correlation direction and edge thickness indicating the absolute Spearman rho value. (**D**) Group-wise distribution of integrated component 1 scores. Pairwise significance was assessed using Wilcoxon rank-sum tests, and only significant comparisons are shown. (**E**) Spearman correlation heatmap between integrated or block-specific component scores and immobility time in the forced swimming test (FST) and tail suspension test (TST). Asterisks indicate nominal significance: **p < 0.01 and ***p < 0.001. CORT, corticosterone; FLX, fluoxetine; BHSST-L, low-dose *Banhasasim-tang*; BHSST-H, high-dose *Banhasasim-tang*. The DIABLO-derived network represents statistical associations among selected variables and does not demonstrate direct biological interactions or causal relationships. These integrated features should be interpreted as hypothesis-generating microbiome–metabolome signatures.

To determine whether BHSST-associated microbial and metabolic changes formed a coordinated multi-omics profile, we performed integrated microbiome–metabolome analysis using block.sPLS-DA within the DIABLO framework, which identifies shared cross-omics features contributing to group discrimination. The microbiome block included LEfSe-selected fecal bacterial taxa, and the metabolome block included annotated differential serum metabolites identified from the CORT versus BHSST-H comparison. The integrated score plot showed separation among the experimental groups, indicating that the selected microbiome and metabolome features jointly captured group-dependent variation (Fig. 5A). The CORT group was clearly separated from the other groups, whereas the FLX and BHSST-treated groups shifted away from the CORT profile.

The loading plot identified the microbiome and metabolome features contributing to integrated component 1 (Fig. 5B). These features included serum metabolites related to aromatic amino acid metabolism, gut microbial co-metabolism, lipid metabolism, and redox-related metabolism, together with fecal bacterial taxa selected by LEfSe. Because loading signs represent the orientation of the latent component, positive and negative loadings were interpreted as opposing feature sets contributing to group discrimination rather than direct biological upregulation or downregulation.

A DIABLO-selected microbiota–metabolite network was then constructed to examine cross-omics associations among the selected features. The network showed significant correlations between a subset of fecal bacterial taxa and serum metabolites, indicating statistical associations between selected microbiome and metabolome features within the integrated model (Fig. 5C).

Finally, we examined the behavioral relevance of the integrated signature. Integrated component 1 scores differed significantly between the CORT group and the Normal, FLX, BHSST-L, and BHSST-H groups (Fig. 5D). In addition, integrated component 1 and metabolome component 1 were negatively correlated with both FST and TST immobility time, while microbiome component 1 was negatively correlated with TST immobility time (Fig. 5E). These findings indicate that the behavioral improvement observed in BHSST-treated mice was accompanied by association-based microbiome–metabolome signatures rather than by a single microbial or metabolic feature. However, these integrated signatures should be interpreted as hypothesis-generating associations and not as evidence of causal biological interactions.

## Discussion

This study showed that BHSST treatment reduced CORT-induced depressive-like behavior and was accompanied by changes in fecal bacterial markers, serum metabolites, and integrated microbiome–metabolome profiles. In the present study, CORT increased immobility time in both behavioral tests, whereas fluoxetine and BHSST reduced these behavioral responses. These findings are consistent with previous reports showing that repeated CORT administration induces depression-like behavior in mice, including increased immobility in the FST and TST [18]. Chronic CORT exposure has also been reported to alter gut microbial composition and metabolomic profiles together with anxiety and depression-related behaviors, supporting the relevance of this model for investigating stress-related gut metabolic changes [19].

The microbiome findings indicate that BHSST affected specific fecal bacterial markers rather than overall microbial diversity. Alpha diversity indices did not differ significantly among groups, suggesting that CORT and BHSST did not markedly alter fecal microbial richness or evenness in this model. This result is consistent with clinical microbiome studies showing that alpha diversity findings in depressive disorder are heterogeneous and not consistently different between patients and controls [20]. In contrast, LEfSe analysis and raw abundance-based post hoc testing identified a subset of bacterial taxa that differed among the experimental groups. These findings suggest that the microbiome response to CORT and BHSST was reflected more clearly in taxon-level differences than in global diversity metrics.

The relationship between gut microbiota and depression has been supported by both clinical and experimental evidence. A systematic review and meta-analysis reported altered gut microbiota composition in depressive disorder, although the results varied across studies and populations [20]. Reviews of gut microbial metabolites in depression further suggest that microbial metabolic functions, rather than taxonomic composition alone, are important for understanding gut–brain communication [21]. Experimental evidence also supports a functional link between depression-associated gut microbiota and host metabolism, as transfer of gut microbiota from patients with major depressive disorder induced depressive-like behaviors and metabolic changes in recipient mice [6]. In addition, recent CORT-based animal work showed that CORT-associated gut dysbiosis could contribute to depressive-like phenotypes through metabolic pathways involving ceramides and mitochondrial dysfunction [22]. In the present study, the CORT group was enriched with Oscillospiraceae, [Eubacterium] siraeum group, Anaeroplasma, and Family XIII AD3011 group. These taxa may provide additional context for CORT-associated taxon-level microbial shifts. Among these taxa, Oscillospiraceae has been reported as one of the major discriminative bacterial families across multiple depressive animal models and was linked to fecal metabolic dysbiosis involving lipid and amino acid metabolism [23]. Anaeroplasma was also reported to be increased in a mouse model showing anxiety- and depression-like behaviors associated with gut microbiota dysbiosis [24]. However, because these previous studies differ from the present CORT-induced model and 16S rRNA sequencing does not provide direct functional information, the CORT-enriched taxa identified in this study should be interpreted cautiously as CORT-associated discriminative markers rather than confirmed microbial contributors to depressive-like behavioral changes.

The BHSST-associated taxa provide biological context for the observed microbiome changes. Among these taxa, *Faecalibaculum rodentium* has been reported to regulate intestinal epithelial homeostasis through retinoic acid signaling and eosinophil-dependent mechanisms [25]. *Lactobacillus* and *Bifidobacterium* have also been investigated in relation to gut–brain communication. For example, *Lactobacillus rhamnosus* has been reported to regulate emotional behavior and central GABA receptor expression through the vagus nerve in mice [26], and *Bifidobacterium longum* reduced depression scores and altered brain activity in patients with irritable bowel syndrome [27]. In addition, *Akkermansia muciniphila* has been discussed as a beneficial mucin-associated bacterium related to gut barrier function and host metabolic regulation [28]. These previous findings support the biological relevance of the taxa identified in the present study. However, because the current analysis was based on 16S rRNA sequencing, it does not provide strain-level resolution or direct functional information. Therefore, these taxa should be interpreted as discriminative bacterial markers associated with BHSST treatment, rather than as confirmed functional mediators. Because microbiome differential abundance results can vary depending on the statistical method and dataset structure [29], the LEfSe-derived taxa were interpreted cautiously. The MaAsLin2 sensitivity analysis showed partial overlap with the LEfSe findings but did not reproduce all marker taxa. Therefore, these taxa should be regarded as exploratory discriminative markers rather than definitive differentially abundant taxa.

Serum metabolomics showed that BHSST-H was associated with metabolic differences in CORT-induced mice. PCA revealed group-dependent metabolic variation in both ion modes, and VIP-based analysis identified annotated metabolites that distinguished CORT from BHSST-H. These metabolites were related to aromatic amino acid metabolism, gut microbial co-metabolism, redox regulation, lipid metabolism, and the citrate cycle. As a multi-herb formula, BHSST may affect multiple biological processes rather than a single molecular target. This interpretation is consistent with network pharmacology evidence suggesting that *Banxia Xiexin* Decoction may be associated with inflammatory response, oxidative stress response, lipid metabolism, serotonergic synapse-related pathways, arachidonic acid metabolism, HIF-1 signaling, and NF-κB signaling [11]. Because that evidence is based on network prediction rather than experimental validation in a CORT-induced depression model, it should be regarded as supportive. An experimental atherosclerosis co-depression model also reported that *Banxia Xiexin* Decoction modulated gut microbiome composition and lipid metabolic profiles, although that disease model differs from the present CORT-induced depression-like model [12].

Several annotated metabolites provide plausible biological context for the observed metabolic changes. Hippuric acid is a host–microbe co-metabolite, and microbial aromatic amino acid metabolism can contribute to systemic hippuric acid production [30]. Indolelactic acid is also relevant to gut–host interaction because recent preclinical evidence showed that *Pediococcus acidilactici* attenuated chronic stress-induced depression through indole-3-lactic acid production and suppression of neuroinflammation [31]. Changes in glutathione and pyroglutamic acid may reflect redox-related metabolic alterations. Glutathione alterations have been reported in major depressive disorder based on proton magnetic resonance spectroscopy studies [32]. Lipid-related metabolites, including LPCs, should be interpreted cautiously, but serum lipidomic alterations have been investigated in patients with major depressive disorder, supporting the relevance of lipid metabolism in depression research [33]. These findings provide biological context for the metabolomics results, although targeted validation using authentic standards is required.

Pathway analysis provided additional context but should be interpreted cautiously. Phenylalanine metabolism, the citrate cycle, glutathione metabolism, glyoxylate and dicarboxylate metabolism, glycerophospholipid metabolism, and phenylalanine, tyrosine, and tryptophan biosynthesis showed nominal enrichment based on raw p-values. However, no pathway passed the FDR *q* < 0.1 threshold. Therefore, these results should not be described as definitive pathway enrichment. Rather, they provide exploratory pathway-level information suggesting that BHSST-H-associated metabolites may be related to amino acid metabolism, mitochondrial energy metabolism, redox regulation, lipid metabolism, and microbial co-metabolism.

The integrated microbiome–metabolome analysis should be interpreted as an exploratory framework linking taxonomic and metabolic changes observed after BHSST treatment. DIABLO/block.sPLS-DA was used because it can identify shared information across different omics blocks while discriminating phenotypic groups [16]. In the present study, the integrated analysis identified association-based cross-omics signatures involving fecal bacterial markers, serum metabolites, and behavioral outcomes. However, the DIABLO-derived network and correlation results represent statistical associations among selected variables, rather than direct biological interactions or causal relationships. Therefore, these integrated features should be regarded as hypothesis-generating signatures rather than validated biomarkers, causal mediators, or mechanistic evidence. Further studies using microbiota manipulation, fecal microbiota transplantation, germ-free or antibiotic-treated models, and targeted metabolite intervention are needed to determine whether the identified taxa or metabolites functionally contribute to the behavioral effects of BHSST.

This study has several limitations. First, the behavioral assessment was based on the FST and TST, which primarily measure immobility-based behavioral despair-like responses and are widely used for antidepressant screening. Therefore, the behavioral effects of BHSST should be interpreted as antidepressant-like effects in these screening tests rather than as comprehensive evidence of recovery from depression-like phenotypes. The microbiome analysis was based on 16S rRNA sequencing, which limits taxonomic resolution and does not directly measure microbial function. In addition, the metabolomics analysis was untargeted; therefore, the annotated metabolites require further confirmation using targeted LC-MS/MS with authentic standards. Finally, the correlation, network, and multi-omics integration analyses indicate associations but cannot establish causality. Despite these limitations, this study showed that BHSST improved CORT-induced depressive-like behavior and was accompanied by changes in fecal bacterial markers, serum metabolic profiles, and integrated microbiome–metabolome signatures. These findings suggest that BHSST treatment is associated with coordinated gut–metabolic changes in a stress-related depression-like model and provide a basis for further investigation of BHSST as a multi-component herbal intervention targeting gut–metabolic pathways.

## Conclusion

In conclusion, BHSST attenuated CORT-induced depressive-like behavior in mice, as demonstrated by reduced immobility time in the FST and TST. This behavioral improvement was accompanied by selective remodeling of fecal bacterial taxa and broad changes in serum metabolic profiles. BHSST-H-associated metabolites were mainly related to amino acid metabolism, energy metabolism, antioxidant-related metabolism, lipid metabolism, and gut microbial co-metabolism, although pathway-level findings should be interpreted as exploratory. Integrated microbiome-metabolome analysis further identified association-based multi-omics signatures linking BHSST-associated microbial and metabolic features with depressive-like behavioral outcomes. These findings suggest that the behavioral effects of BHSST are associated with coordinated microbiome–metabolome changes rather than with a single microbial or metabolic marker. Because these integrated signatures are hypothesis-generating, further causal and targeted validation studies are required to confirm the biological relevance of the identified taxa and metabolites.

## Supplemental Materials

Supplementary data for this paper are available on-line only at http://jmb.or.kr.
