# Supplementary material for: Banhasasim-tang Attenuates Corticosterone-Induced Behavioral Despair-like Responses and Is Associated with Gut Microbiota and Serum Metabolomic Changes in Mice
Source: J Microbiol Biotechnol. 2026 Aug 4;36:e2606018. doi: 10.4014/jmb.2606.06018 (PMC13526893; doi:10.4014/jmb.2606.06018)
Supplement: Supplementary file 1 [file jmb-36-e2606018-supple.zip › jmb-36-e2606018-supple.pdf]

J. Microbiol. Biotechnol. 2026. 36: e2606018

https://doi.org/10.4014/jmb.2606.06018

Supplementary Figure

Fiber-Type Invasive Laser Acupuncture Modulates Structural, Biochemical, and Serum Metabolomic Responses in Monosodium Iodoacetate-Induced Knee Osteoarthritis Rats

J. Microbiol. Biotechnol. 2026. 36: e2606018

https://doi.org/10.4014/jmb.2606.06018

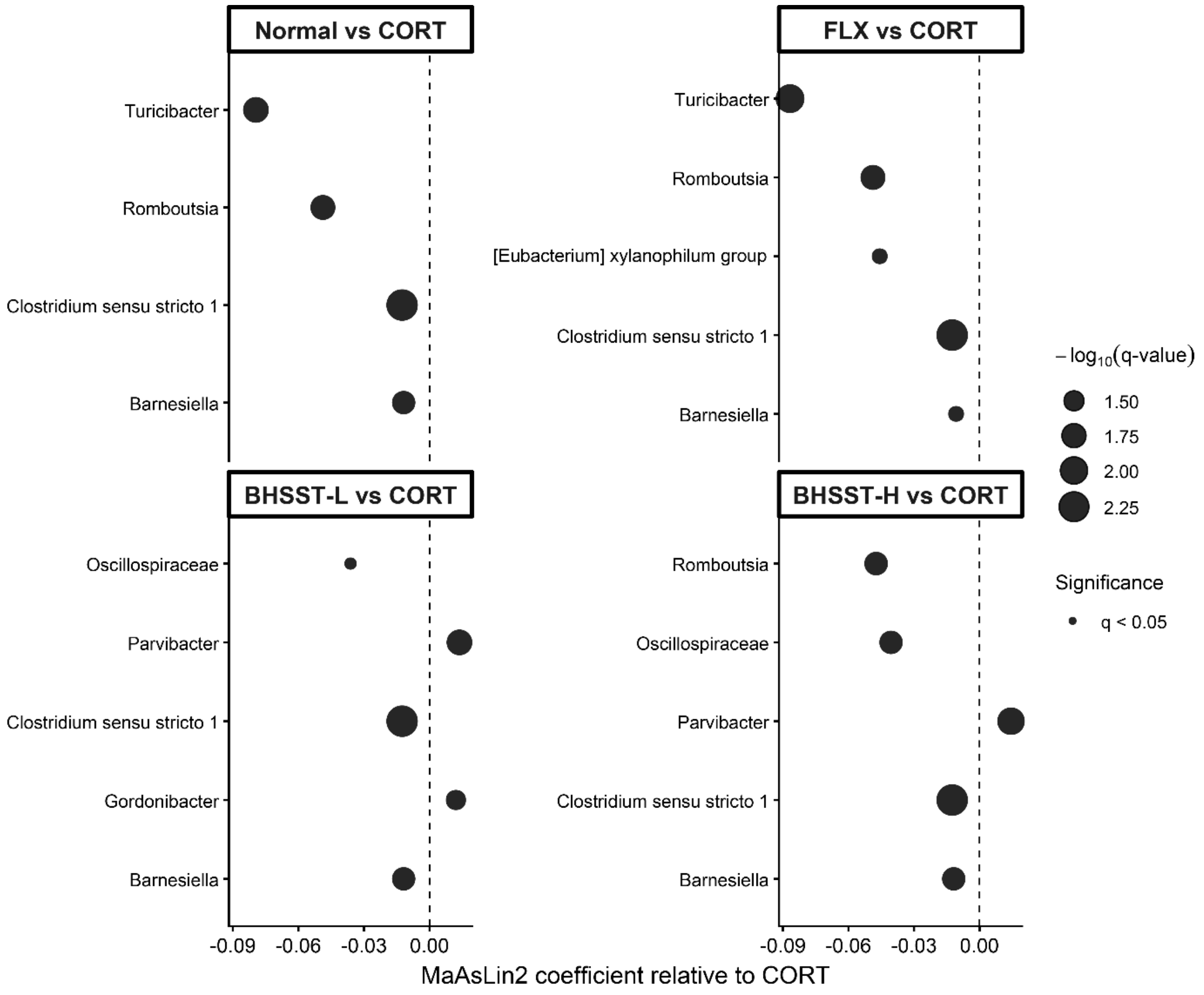

Fig. S1. MaAsLin2 sensitivity analysis of genus-level fecal bacterial taxa. MaAsLin2 was performed using genus-level relative abundance data after arcsine square-root transformation. The CORT group was used as the reference group. The coefficient indicates the direction and magnitude of group-associated differences relative to CORT. Positive coefficients indicate higher abundance than CORT, whereas negative coefficients indicate lower abundance than CORT. Point size represents −log10(q-value). Taxa with $q < 0.05$ are displayed. CORT, corticosterone; FLX, fluoxetine; BHSST-L, low-dose Banhasasim-tang; BHSST-H, high-dose Banhasasim-tang.
